# Long-Term Management of Sleep Apnea-Hypopnea Syndrome: Efficacy and Challenges of Continuous Positive Airway Pressure Therapy—A Narrative Review

**DOI:** 10.3390/medsci13010004

**Published:** 2024-12-30

**Authors:** Zishan Rahman, Ahsan Nazim, Palvi Mroke, Khansa Ali, MD Parbej Allam, Aakash Mahato, Mahveer Maheshwari, Camila Sanchez Cruz, Imran Baig, Ernesto Calderon Martinez

**Affiliations:** 1 Department of Medicine, Caribbean Medical University, Rosemont, IL 60018, USA; zishanrahman922@outlook.com (Z.R.); pmroke93@gmail.com (P.M.); 2 Department of Medicine, Liaquat University of Medical and Health Sciences, Jamshoro 76060, Pakistan; ahsanparacha35@gmail.com (A.N.); khansaalijatoi@gmail.com (K.A.); mahveermaheshwari10@gmail.com (M.M.); 3 Department of Medicine, Kathmandu Medical College, Kathmandu 44600, Nepal; parbejkhan2013@gmail.com; 4 Department of Medicine, BP Koirala Institute of Health Sciences, Dharan 56700, Nepal; aakashmahatobrt@gmail.com; 5 Department of Medicine, Universidad Nacional Autonoma de México (UNAM), Mexico City 04510, Mexico; c.camilasc1@gmail.com; 6 Houston Methodist West Hospital, Houston, TX 77094, USA; imranbaigmd@gmail.com

**Keywords:** continuous positive airway pressure, obstructive sleep apnea, central sleep apnea, sleep apnea hypopnea syndrome, CPAP adherence, CPAP noncompliance, apnea hypopnea index, bilevel positive airway pressure, adaptive servo ventilation, risk factors for obstructive sleep apnea

## Abstract

Sleep apnea-hypopnea syndrome (SAHS) is a respiratory disorder characterized by cessation of breathing during sleep, resulting in daytime somnolence and various comorbidities. SAHS encompasses obstructive sleep apnea (OSA), caused by upper airway obstruction, and central sleep apnea (CSA), resulting from lack of brainstem signaling for respiration. Continuous positive airway pressure (CPAP) therapy is the gold standard treatment for SAHS, reducing apnea and hypopnea episodes by providing continuous airflow. CPAP enhances sleep quality and improves overall health by reducing the risk of comorbidities such as hypertension, type 2 diabetes mellitus, cardiovascular disease and stroke. CPAP nonadherence leads to health deterioration and occurs due to mask discomfort, unsupportive partners, upper respiratory dryness, and claustrophobia. Technological advancements such as auto-titrating positive airway pressure (APAP) systems, smart fit mask interface systems, and telemonitoring devices offer patients greater comfort and enhance adherence. Future research should focus on new technological developments, such as artificial intelligence, which may detect treatment failure and alert providers to intervene accordingly.

## 1. Introduction

Sleep apnea-hypopnea syndrome (SAHS) is a respiratory disorder characterized by interrupted breathing during sleep, resulting in daytime somnolence, among other symptoms [1]. SAHS encompasses a multitude of sleep-related breathing disorders, including obstructive sleep apnea (OSA), central sleep apnea (CSA), and mixed sleep apnea [2,3]. OSA is caused by upper airway obstruction during sleep, often due to obesity [3,4]. The rising global prevalence of OSA, estimated at approximately 1 billion individuals aged 30 to 65, is mainly due to increasing obesity rates, defined as a body mass index (BMI) greater than 30. Additional risk factors include neck circumference greater than 17 inches, males over 50 years old, and anatomical abnormalities. OSA increases the risk of type 2 diabetes mellitus, hypertension, and cardiovascular disease [4,5]. Due to the sleep impairment caused by OSA, patients may experience cognitive decline and depression and are at a greater risk of motor vehicle accidents [6]. Conversely, CSA results in impaired respiration due to brain stem dysfunction [7,8]. CSA accounts for 5–10% of sleep breathing disorders and is more prevalent in patients who have had a stroke [9,10]. A 2016 study found that the prevalence of CSA in heart failure patients was approximately 0.9%, while OSA was significantly more common, affecting 55.1% [11]. CPAP has demonstrated benefits in both CSA and OSA by reducing the risk of arrythmia [12]. Mixed sleep apnea, or complex sleep apnea, is the simultaneous presence of both OSA and CSA. The combination of obstruction and lack of stimulatory input for respiration makes mixed sleep apnea challenging to manage [13].

This article aims to provide an in-depth review on the effectiveness of CPAP therapy in managing SAHS and its impact on cardiovascular, metabolic, and cognitive health. Additionally, alternative treatment approaches to CPAP are compared, such as oral appliances, positional therapy, and surgical interventions. We outline the barriers of CPAP nonadherence and the benefits of lifestyle modifications like weight loss and smoking cessation, which may contribute to an improved quality of life for patients with SAHS.

## 2. Diagnostic Criteria and Tools

Clinical evaluation of SAHS includes reviewing the patient’s medical history, performing a physical examination, and assessing symptoms such as snoring, nighttime awakenings, fatigue, and choking [14,15]. Diagnostic testing should be considered for non-specific symptoms, such as unexplained nocturia, difficulty concentrating, poor sleep quality, and paroxysmal nocturnal dyspnea, particularly in patients with CSA [16]. A medical history of obesity, smoking, diabetes, hypertension, and opioid use are commonly associated with OSA. One study identified an increased prevalence of Hashimoto thyroiditis among females with severe OSA. While there is no significant correlation between thyroid volume and severity of OSA, isthmus thickness was significantly correlated with the apnea-hypopnea index (AHI) [15,17]. CSA is noted in association with OSA, heart failure, atrial fibrillation, cerebrovascular accident tetraplegia, and chronic opioid use [8].

Physical examination findings that may suggest the presence of OSA include a neck circumference exceeding 17 inches in men or 16 inches in women, a BMI over 30, and a Friedman tongue position class 3 or higher. Additional indicators of OSA are oral and maxillofacial features such as enlarged tonsils, macroglossia, jaw misalignment, an overbite where the upper teeth protrude beyond the lower teeth, and an overjet where the upper teeth extend significantly beyond the lower teeth. Nasal abnormalities like turbinate hypertrophy and a deviated septum can also increase the risk of OSA [18].

Diagnostic testing for OSA should be combined with a comprehensive sleep evaluation and adequate follow-up. Polysomnography (PSG) is considered the definitive standard for diagnosing OSA. In certain cases, the Home Sleep Apnea Test (HSAT) can be used as an initial sleep study in uncomplicated adult patients with signs and symptoms indicating an increased risk of OSA. However, HSAT results do not provide a sufficient post-test likelihood of confirming or ruling out OSA. In addition to diagnosing OSA, PSG can detect several types of sleep-disordered breathing, which makes it the preferred primary diagnostic test [19].

### 2.1. Diagnostic Criteria for Obstructive Sleep Apnea

A diagnosis of OSA can be established through PSG or HSAT testing. Two guidelines published by the American Academy of Sleep Medicine entitled “Practice Parameters for the Indications for Polysomnography and Related Procedures: An Update for 2005” and “Clinical Guidelines for the Use of Unattended Portable Monitors in the Diagnosis of Obstructive Sleep Apnea in Adult Patients (2007)” address the diagnosis of OSA. The American Academy of Sleep Medicine has tasked a group of experts with updating the clinical practice guideline on diagnosing OSA. The new guideline aims to integrate and update prior recommendations on when to use in-laboratory PSG versus HSAT.

A diagnosis can be confirmed if the test reveals either ≥15 predominantly obstructive respiratory events per hour of sleep or ≥5 predominantly obstructive respiratory events per hour of sleep accompanied by at least one of the following: daytime somnolence, non-rejuvenating sleep, lethargy, or insomnia; dyspnea upon awakening, gasping for air, or choking; loud snoring, respiratory halts, or both; or the presence of comorbid illnesses such as hypertension, mood disorders, cognitive dysfunction, coronary artery disease, cerebrovascular accident, heart failure, atrial fibrillation, or type 2 diabetes. Table 1 presents the diagnostic criteria for OSA [19,20].

### 2.2. Diagnostic Criteria for Central Sleep Apnea

The diagnosis of primary CSA is confirmed when PSG shows at least five central apneas and/or central hypopneas per hour during sleep. Furthermore, these events should be for more than half of all respiratory events in the AHI, with no Cheyne–Strokes breathing. In addition, there must be at least one sleep-related complaint, such as drowsiness during the day, insomnia, dyspnea upon awakening, snoring, or observed apneas. To diagnose CSA with Cheyne–Strokes breathing, the primary CSA criteria must be met, as well as three or more consecutive central apneas or hypopneas with a crescendo–decrescendo respiratory rhythm lasting at least 40 s. Treatment-emergent CSA is diagnosed when a primary OSA diagnosis (characterized by an AHI of at least five obstructive events per hour) is followed by the resolution of obstructive apneas and the emergence or persistence of CSA during positive airway pressure titration, without other disease or substance explanations. Table 2 presents the diagnostic criteria for CSA [16].

The AHI is a metric used to assess the severity of sleep apnea. AHI consists of the total number of apnea and hypopnea episodes arising in every hour of sleep. Apnea is a respiratory event resulting in a complete lack of airflow, measured by a greater than 90% reduction in the thermal sensor for 10 or more seconds on PSG. According to the American Academy of Sleep Medicine, hypopnea is characterized by a reduction in nasal pressure of at least 30% for 10 s or more, accompanied by a 3% or greater drop in oxygen saturation or electroencephalogram arousal on PSG [14,21]. A diagnosis of OSA can be established with an AHI value greater than 5 if symptoms are present or an AHI of 15 or more, regardless of symptoms. A diagnosis of OSA should consider the severity (mild, moderate, or severe) to determine whether a patient needs to be treated. The AHI can also be used to categorize the severity of the condition, with a score of 5–14 indicating mild, 15–30 moderate, and 30 or higher severe OSA [22]. Recent evidence has pointed out the use of point-of-care ultrasound to identify parameters to diagnose OSA, such as distance between lingual arteries and mean resting tongue thickness, among others, but these parameters are still not validated and will require the training of physicians for their implementation [23,24,25,26]. The most frequent initial step in the treatment of mild OSA is behavioral or lifestyle modification, including weight loss, alcohol avoidance, and smoking cessation. Additionally, sleep aids such as benzodiazepines and muscle relaxants should be avoided, as they cause can contribute to airway narrowing. Weight loss in overweight patients has been shown to decrease apneic episodes by reducing neck fat and nasopharyngeal crowding. The first-line therapy for moderate to severe sleep apnea is CPAP [14,21].

## 3. Impact of CPAP Therapy on Sleep Quality, Daytime Sleepiness, and Cognitive Function

CPAP therapy significantly improves sleep quality and reduces daytime sleepiness in individuals with OSA but does not have the same effect in CSA. The mechanism of CPAP therapy involves maintaining an open airway through a steady flow of air delivered via a mask, preventing the airway obstruction responsible for apnea and hypopnea. This results in more consistent, uninterrupted sleep with extended restorative periods, including deep and REM sleep [27]. Enhanced sleep architecture is achieved through reduced nighttime awakenings and normalized sleep stages. In patients with moderate to severe OSA, significant improvements in sleep quality and a reduction in sleepiness have been observed, as indicated by a dose-response effect on the Epworth drowsiness scale, a validated instrument for evaluating daytime drowsiness associated with sleep issues [28,29].

Under CPAP, patients report feeling more alert and less fatigued and experience better cognitive function, including enhanced attention, memory, and problem-solving skills. Additionally, CPAP therapy contributes to better mood stability and emotional well-being, mitigating the irritability and mood disorders commonly associated with poor sleep. Patients report improved sleep quality and daytime functioning within one month of CPAP use, with continued enhancements observed at three months [30].

CPAP therapy markedly enhances cognitive abilities, such as attention and memory. After six months of treatment, improvements in cognitive performance and sleep spindle densities were reported, reflecting enhanced mental function [31]. Real-world evidence shows that patients adhering to CPAP experience increased mental clarity, improved memory, and reduced depression and anxiety symptoms. CPAP effectively lowers the AHI and enhances overall cognitive performance and mood [32]. Similarly, CPAP reduces self-reported fatigue in stroke patients with OSA, which improves stroke therapy adherence [33]. Studies have demonstrated that consistent CPAP use in stroke patients with OSA alleviates tiredness and may contribute to better cognitive function, mood, and overall recovery outcomes. This makes CPAP therapy an important consideration in the management of stroke patients with coexisting sleep apnea [34].

### 3.1. Impact on Hypertension and Cardiovascular Outcomes

OSA significantly increases the risk of hypertension and other cardiovascular diseases due to intermittent hypoxia and elevated sympathetic nervous system activity, which is caused by recurrent apneas during sleep. CPAP is an effective treatment for reducing blood pressure in patients with OSA, particularly those with treatment-resistant hypertension. Studies have demonstrated that blood pressure reductions can occur within weeks of starting CPAP therapy [35]. Within 1–3 months, many patients experience noticeable decreases in daytime and nighttime blood pressure [36]. Continued use of CPAP over 3 to 6 months generally leads to further reductions and stabilization of blood pressure [37]. The most significant blood pressure reductions are often observed during this period, especially among patients who use CPAP consistently each night. CPAP therapy improves blood pressure patterns, transitioning patients from a “non-dipping” to a “dipping” nocturnal pattern, in which the pattern indicates a blood pressure drop of 10%, which reduces the risk of hypertension. It also improves pulmonary hypertension by augmenting pulmonary vascular responsiveness to hypoxia [38,39]. For individuals with both coronary artery disease and moderate to severe OSA, CPAP therapy can help prevent all-cause mortality and reduce major adverse cardiovascular events. Additionally, consistent nightly usage of CPAP for an average of 4 h reduces the need for revascularization, enhancing both immediate and long-term cardiovascular outcomes [40,41].

Lifestyle changes, particularly weight loss, can significantly reduce the risk of cardiovascular events in obese patients and are recommended for all individuals with OSA. While primarily used to manage OSA, CPAP therapy can also contribute to weight control through several mechanisms. CPAP therapy improves leptin resistance and alters ghrelin plasma levels, which helps enhance hunger and appetite control [42]. Additionally, CPAP therapy increases daytime vigilance by improving sleep quality and reducing daytime sleepiness, enabling patients to be more physically active. This increased activity can further support weight management and cardiovascular health [43].

### 3.2. Impact on Metabolic Parameters

OSA is associated with type 2 diabetes and disrupted glucose metabolism. A systematic review and meta-analysis revealed that CPAP treatment significantly reduced hemoglobin A1c by 0.24% compared to inactive controls [44]. Increased severity of sleep apnea is associated with an increased risk of diabetes, and the risk may be partially explained by hypoxemia and arousal. In OSA, intermittent hypoxia during sleep is considered an important stimulus that leads to insulin resistance because it triggers the sympathetic nervous system, increasing stress hormone levels like cortisol and catecholamines, which can promote insulin resistance [45]. Hence, as a treatable condition, sleep apnea may represent a modifiable risk factor for the development of diabetes [46]. Moreover, studies also highlight that OSA is associated with increased inflammatory markers, including C-reactive protein, linked to insulin resistance and an increased risk of cardiovascular disease. Hence, CPAP therapy has been shown to reduce these inflammatory markers [47].

As obesity and dyslipidemia often coexist with OSA, by lowering oxidative stress, improving hyperglycemia, aiding in weight loss, as discussed earlier, and lowering total cholesterol, CPAP can potentially aid in managing metabolic syndrome [48,49,50,51]. However, some studies that analyzed lipid profiles as a secondary outcome observed no significant lipid-lowering effect of CPAP. The effect of CPAP treatment on lipid profiles in OSA is yet to be understood [52]. Some new correlations have shown that OSA can be related to vitamin D deficiency, suggesting vigilance regarding these metabolic parameters [53,54]. In addition to CPAP, weight loss medications for diabetes, such as tirzepatide, have demonstrated success in the management of metabolic dysfunction in OSA patients. A randomized, double-blinded study conducted over 52 weeks revealed that tirzepatide reduced inflammatory markers such as C-reactive protein and lowered blood pressure. Tirzepatide significantly reduced the AHI at 52 weeks, compared to placebo, with a mean reduction of 20 events per hour in patients not using positive airway pressure therapy and 23.8 events per hour in patients using positive airway pressure therapy, highlighting its potential to synergistically improve OSA when combined with CPAP therapy [55].

### 3.3. Neurocognitive Benefits

Research has demonstrated that CPAP therapy can significantly enhance aspect of cognitive function, such as vigilance, attention, memory, and executive functioning [56]. This is particularly important for individuals with OSA, who often suffer from deficits in these cognitive domains due to disrupted sleep architecture and intermittent hypoxia. In patients with Alzheimer’s disease, therapeutic trials of treatment with CPAP have been shown to slow or even improve cognitive impairment [57,58]. Furthermore, CPAP has been demonstrated to increase grey matter volume in the hippocampal and frontal regions [59]. OSA is associated with poor sleep quality, which can contribute to mood disorders like depression and anxiety. By improving sleep quality and oxygen levels, CPAP therapy can help reduce the severity of these mood disorders. Hence, CPAP should be the first choice of treatment before starting other treatments for depression and anxiety symptoms [60]. CPAP therapy also improves the physical rehabilitation process in stroke patients [61].

### 3.4. Adherence to CPAP Therapy

Consistent use of CPAP therapy is necessary for successfully managing SAHS, as inadequate adherence can result in continuous breathing disruptions, exacerbated symptoms, and increased comorbidities. Nevertheless, CPAP adherence can be challenging to maintain due to multiple factors. Discomfort from CPAP treatment can occur due to improper mask fitting and skin irritation. CPAP may also cause nasal congestion for up to 45–69% of users due to irritation caused by airflow [62]. Furthermore, claustrophobia is a common obstacle in CPAP adherence, causing patients to experience fear of suffocation and restriction [63]. Additional contributing factors for nonadherence to CPAP include higher AHI and an unsupportive bed partner [64,65].

CPAP adherence can be enhanced by guiding patients on proper device use to prevent discomfort. Implementing desensitization techniques and cognitive behavioral therapy reduces nonadherence due to claustrophobia [62,63,64]. Using nasal saline sprays may improve nasal congestion and skin irritation, which can be relieved by petroleum-free skin moisturizers. Recent technological advancements have improved adherence to CPAP therapy. Mask liners and humidification devices reduce dry air from irritating the nasal passage, further decreasing mucosal edema and nasal congestion [64].

Adherence within the first week of CPAP use is a strong indicator of long-term success, making early assessment of risk factors for nonadherence crucial. During the initial two weeks, patients develop opinions on OSA and its treatment, making their perceptions critical to adherence. Although the American Academy of Sleep Medicine recommends patient education to improve adherence, evidence suggests that education alone may not fully address adherence challenges. Therefore, adherence is influenced by environmental, motivational, and psychological factors, in addition to technological aspects [65].

### 3.5. Side Effects and Complications

CPAP therapy can cause problematic side effects, such as nasal congestion, sore throat, and dryness of the nose and throat. Mask leaks may increase nasal resistance and worsen symptoms. Heated humidifiers provide air moisture, reducing nose and throat dryness. By reducing side effects and increasing comfort, humidifiers ultimately increase adherence. However, humidifiers have drawbacks, such as condensation build-up, disturbing loud noises, and extensive cleaning maintenance requirements [66].

Dermatologic complications may arise with CPAP therapy due to contact irritation between the mask and the skin. Pressure sores may also develop in response to high air pressure and tight masks. Pressure maintained at 35 mmHg or higher for over 2 h can cause irreversible ischemia and tissue necrosis. Allergic contact dermatitis can occur due to diallyl thioureas in neoprene mask straps. Using cloth straps instead may help prevent the development of chronic contact dermatitis [67,68].

CPAP-induced aerophagia is a concerning side effect involving air swallowing, resulting in significant abdominal distention and distress. Aerophagia has been found to exacerbate symptoms of gastroesophageal reflux disease (GERD) further. Combating aerophagia-induced GERD has been successful in cases where upper esophageal sphincter pressure is increased. Baclofen reduces lower esophageal sphincter tone, resulting in less frequent aerophagia-induced GERD [69].

Furthermore, CPAP use may also contribute to the development of keratoconjunctivitis sicca, commonly known as dry eye disease. Improper fitting of CPAP masks often results in air leaks, leading to eye dryness and irritation. Using individually fitted masks instead of standard masks may improve dry eyes by reducing air leakage. Lubricating eye drops and blinking exercises may also provide relief. Individuals with a history of keratopathy, recent eye surgery, or other inflammatory or autoimmune conditions should be carefully monitored, as these conditions can further worsen dry eyes and associated symptoms [70].

### 3.6. Special Populations

Elderly patients often face challenges in utilizing CPAP therapy for sleep apnea due to cognitive decline and physical limitations. This can negatively impact treatment adherence and efficacy. Cognitive impairments, such as those seen in Alzheimer’s disease and other forms of dementia, can complicate CPAP use. Patients with cognitive decline may struggle with the device’s mechanics or may not understand its importance, leading to lower adherence rates [71]. Physical dexterity and upper extremity strength often deteriorate with age, making using CPAP equipment difficult. This includes having trouble donning and adjusting the CPAP mask, leading to discomfort and lower therapy compliance [72]. To address the challenges of CPAP therapy in elderly patients with comorbidities, including physical limitations and cognitive impairments, healthcare providers should implement several strategies. First, they should offer customized instructions by providing clear and concise instructions for using CPAP while accounting for potential cognitive impairments [73].

Additionally, physical modifications should be considered. For individuals with arthritis or limited dexterity, it is beneficial to suggest CPAP masks and equipment that are simpler to use [74]. Finally, therapy regimens should be tailored to patients’ needs by adjusting CPAP settings and apparatuses to accommodate their unique requirements and constraints [75].

Implementing CPAP therapy for disorders like OSA can be difficult for individuals with comorbidities, such as dialysis patients. Further obstacles, including fluid imbalance or cardiovascular problems, can arise in dialysis patients and impact CPAP treatment. It can be difficult to fit CPAP therapy around dialysis appointments. Long dialysis sessions can interfere with sleep cycles, which makes using CPAP consistently more difficult. To address these comorbidities, various strategies should be taken. To guarantee a coordinated strategy to manage dialysis and CPAP therapy, nephrologists, sleep specialists, and other healthcare providers should work together. They should plan frequent follow-ups to assess the patient’s comfort and the therapy’s effectiveness. They should also utilize information from CPAP devices to monitor adherence and make required modifications [76].

Children’s and adolescents’ distinct physiological, developmental, and psychological needs necessitate specific consideration when administering CPAP therapy. Compared to adults, children’s airways are more flexible and smaller. As children grow, their needs for CPAP may also alter. Therapy must be routinely reevaluated to account for changes in body size and airway structure. This calls for modifying the mask fit and CPAP pressure settings. In addition, adolescents may be vulnerable to peer attitudes and societal perceptions. Their inclination to use CPAP regularly may be impacted by the sight of the apparatus, which can be embarrassing. To help children and adolescents build coping mechanisms and positive attitudes regarding CPAP therapy, cognitive behavioral therapy and other behavioral interventions can be useful in managing adherence concerns [77].

### 3.7. Alternative and Adjunctive Therapies

**Figure 1:**
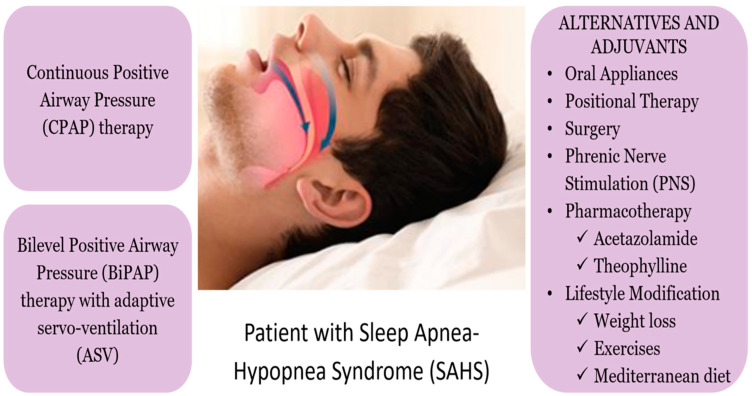
CPAP therapy alternatives and adjuvants for patients with SAHS. Abbreviations: SAHS—sleep apnea hypopnea syndrome, CPAP—continuous positive airway pressure, BiPaP—bilevel positive airway pressure, ASV—adaptive servo-ventilation, PNS—phrenic nerve stimulation. Figure created with BioRender. All credits to Aakash Mahato.

CPAP therapy significantly improves sleep quality and reduces daytime sleepiness in individuals with OSA but is not as effective for CSA [27]. CPAP’s constant pressure that maintains open airways does not address the shallow breathing and breathing pauses of CSA and Cheyne–Stokes breathing. On the other hand, bilevel PAP (BiPAP) therapy with adaptive servo-ventilation changes the pressure based on the patient’s needs to treat CSA [78]. Figure 1 displays the alternative therapies for OSA, in addition to APAP.

Oral appliances are frequently prescribed as a non-invasive treatment option for patients with OSA. The most common device is a custom-made mandibular advancement device (MAD), which gradually moves the lower jaw forward. While CPAP is better at reducing sleep apnea symptoms, research shows that health outcomes with MAD are similar to those with CPAP, with some studies demonstrating that patients are more likely to use MAD consistently [79,80]. MAD increases the volume of the upper airway, reducing the likelihood of airway narrowing or collapse. Several studies have recommended using MAD for treating mild to moderate OSA and have also indicated its use in patients with severe OSA who cannot tolerate CPAP [81].

Some patients with sleep apnea experience worsened breathing when lying on their back, a condition known as positional obstructive sleep apnea, while others may only have issues in the supine position, referred to as exclusive positional obstructive sleep apnea. These individuals often comply with CPAP treatment and might use devices that keep them from lying on their back by vibrating their neck or chest. Studies show these devices can help, but not as much as CPAP. They work well in the short term, but only 41.6% of people use them after six months. They are more helpful for people with mild to moderate sleep apnea than for those with severe sleep apnea [82].

Surgical options, such as maxillofacial or head and neck surgery, offer various treatments for OSA patients, especially those who do not respond to conservative therapy, are unable to use CPAP, or have severe symptoms. These surgical options include nasal airway surgery like septoplasty and rhinoplasty, throat surgery like uvulopalatopharyngoplasty, laser-assisted uvulopalatoplasty, and other procedures to shorten or stiffen the palate. Other procedures include hyoid myotomy and suspension, glossectomy, genial tuberosity advancement, jaw surgeries like maxillomandibular advancement or bimaxillary osteotomy, and tracheostomy. These surgeries, especially uvulopalatopharyngoplasty/laser-assisted uvulopalatoplasty with genioglossus advancement and maxillomandibular advancement, can be highly successful, with success rates over 85% and some cases showing results in the high 90% range [83].

Pharmacologic use of acetazolamide has shown improvement in both CSA and OSA. More research is required to determine if it is effective for long-term treatment [84]. Theophylline helps treat CSA and periodic breathing, but its effects on OSA are mixed. It does not add any short-term benefits for apnea, oxygen levels, or breathing pressures once CPAP therapy is in place for mild to moderate OSA [85]. Studies on medications like liraglutide and gliflozins have been performed in diabetic OSA patients. These drugs may help lower heart and metabolism risks, in addition to helping with weight loss [82].

Additionally, studies have shown that transvenous phrenic nerve stimulation significantly reduces the severity of CSA and improves sleep, daytime sleepiness, heart function, symptoms, and quality of life for up to 12 months. This new treatment is safe, effective, and works regardless of patient adherence [86].

The American Thoracic Society recommends lifestyle modifications for OSA patients, such as joining a comprehensive lifestyle program that includes exercise, weight loss, and a Mediterranean diet. Since OSA is common in people who are overweight or obese, and obesity is a major risk factor for OSA, losing weight can help reduce OSA severity. A study performed with the Wisconsin Sleep Cohort found that a 10% weight gain increases AHI by 32%, while a 10% weight loss decreases it by 26%. Therefore, weight loss should be recommended for overweight or obese OSA patients, even though it rarely cures OSA completely. For severely obese patients who struggle to lose weight, bariatric surgery evaluation is suggested if there are no contraindications [82].

While weight loss is the most obvious reason exercise might reduce OSA severity, studies show that exercise can lower AHI even without weight loss. Exercise might help with OSA by strengthening breathing and throat muscles, reducing sleep disruptions, decreasing nasal resistance, and preventing fluid buildup in the legs [87]. Following a Mediterranean diet and staying active did not show a strong link to SAHS alone. However, these measures had a small positive effect on minimum oxygen saturation levels. Eating red meat was linked to a moderate risk of developing SAHS [88].

## 4. Future Directions

Advancements in CPAP therapy have revolutionized the management of SAHS. Patients often discontinue CPAP treatment if they experience discomfort. With new technological advancements, patients are more likely to adhere to CPAP therapy when they experience minimal discomfort, fewer side effects, and increased convenience. Proper mask fitting, auto-adjusting positive airway pressure (APAP), and integrated telemonitoring systems contribute to this improved adherence. The Smart Fit CPAP system utilizes three-dimensional scans to suggest a precise patient mask fit. This system significantly improves adherence by minimizing limiting factors such as air leaks and reducing skin irritation, pressure sores, and upper respiratory dryness. The customized fit prevents overtightening, ensuring comfort and promoting consistent use [89]. The introduction of auto-titrating machines has greatly improved CPAP adherence by enhancing comfort and reducing the need for manual adjustments. While CPAP machines deliver constant pressure, APAP can precisely adjust air pressure levels in response to apneic or hypopnea episodes [90]. Integrated telemonitoring systems such as wearable devices allow the detection of key vital signs such as body temperature, heart rate, and blood oxygen levels. These systems enhance adherence by supplying patients with valuable information, which boosts motivation and encourages greater participation in self-care [91]. Overall, increased adherence and consistent CPAP use lead to better treatment efficacy and health outcomes [28].

### 4.1. Limitations

Research on CPAP therapy may have various limitations that obstruct a deeper understanding of its effectiveness and barriers. We recommend that long-term adherence studies be performed to better understand compliance rates with CPAP therapy. Also, studies on the impact of cognitive decline or psychiatric illnesses on CPAP adherence should be established, since there is little research reviewing these data. The effect of CPAP therapy on different race and gender groups is also not fully understood, and further research should be performed investigating potential variables. Additionally, there are gaps in research concerning patient outcomes, quality of life, and daily functioning based on data collected over extended periods.

### 4.2. Future Recommendations

Future research on CPAP management for SAHS may focus on multiple areas to further enhance patient care and outcomes. The development of artificial intelligence technology with electronic health record systems can notify healthcare providers when a patient is at risk of apneic episodes, but training medical providers to use these tools is needed [92]. Furthermore, AI can detect treatment failure and notify providers to intervene promptly to optimize care. Incorporating AI into clinical workflows can increase efficiency and enable providers to manage a greater patient load more effectively [93]. Large-scale randomized controlled trials and analysis should be conducted to assess the efficacy and safety of new treatments and technologies for SAHS [94]. Additionally, over several years, longitudinal studies can evaluate the long-term outcomes of sleep apnea treatment methods, including CPAP, BiPAP, and surgical interventions.

## 5. Conclusions

CPAP therapy is the most effective treatment in the management of OSA. The research reviewed in this article highlights the positive outcomes associated with CPAP’s ability to reduce apnea and hypopnea episodes. Implementing CPAP results in improved sleep quality, decreased daytime sleepiness, and enhanced cognitive function. The risk of developing mood disorders, including major depressive disorder, is also reduced. Long-term CPAP use is effective in treating treatment-resistant hypertension and lowers the risk of developing major adverse cardiovascular events, such as myocardial infarction and stroke.

Furthermore, consistent adherence to CPAP therapy strengthens the management of type 2 diabetes mellitus by enhancing long-term glycemic control. Despite the advantages of CPAP therapy, challenges such as mask discomfort, nasal congestion, and feelings of claustrophobia can negatively impact adherence. Advancements such as APAP machines and improved mask fitting help increase adherence and comfort. Challenges remain with nonadherence in elderly patients with cognitive impairments and in patients with conditions that require dialysis. A personalized, patient-centered approach should be implemented to enhance the quality of life for patients undergoing CPAP therapy. Patient concerns about discomfort, nasal congestion, and psychological barriers should be addressed through education, cognitive behavioral therapy, and consistent follow-up care. Further research on the use of artificial intelligence may improve CPAP management by notifying providers of apneic episodes.

## Figures and Tables

**Table 1:** Diagnostic Criteria for Obstructive Sleep Apnea.

Obstructive Sleep Apnea Polysomnogram (PSG) and Home Sleep Apnea Test (HSAT) Findings:
(A) ≥15 predominantly obstructive respiratory events every hour OR
(B) ≥5 predominantly obstructive respiratory events every hour and at least one of following:
Daytime somnolence, non-rejuvenating sleep, lethargy, or insomnia
Dyspnea upon awakening, gasping, or choking
Loud snoring, respiratory halts, or both
Hypertension, mood disorder, cognitive dysfunction, coronary artery disease, cerebrovascular accident, heart failure, atrial fibrillation, or diabetes type 2

**Table 2:** Diagnostic Criteria for Primary Central Sleep Apnea.

Primary Central Sleep Apnea Polysomnogram (PSG) Findings:
(A) ≥5 central apneas and/or central hypopneas every hour of sleep, with central events constituting more than half of the total respiratory events and no evidence of Cheyne–Stokes breathing
(B) At least one sleep-related symptom such as: drowsiness during the day, insomnia, dyspnea upon awakening, snoring, or observed apneas

## Data Availability

The data used in this narrative review are publicly available and can be accessed through the references cited in this manuscript.

## Abbreviations

AHI (apnea-hypopnea index), APAP (auto-titrating positive airway pressure), BiPAP (bilevel positive airway pressure), BMI (body mass index), CPAP (continuous positive airway pressure), CSA (central sleep apnea), GERD (gastroesophageal reflux disease), HSAT (home sleep apnea test), MAD (mandibular advancement device), OSA (obstructive sleep apnea), PSG (polysomnography), SAHS (sleep apnea-hypopnea syndrome).
